# Activated Human T Cells Secrete Exosomes That Participate in IL-2 Mediated Immune Response Signaling

**DOI:** 10.1371/journal.pone.0049723

**Published:** 2012-11-16

**Authors:** Jessica Wahlgren, Tanya De L. Karlson, Pernilla Glader, Esbjörn Telemo, Hadi Valadi

**Affiliations:** 1 University of Gothenburg, Department of Rheumatology and Inflammation Research, Gothenburg, Sweden; 2 University of Gothenburg, Respiratory Medicine and Allergology, Gothenburg, Sweden; Institute of Cancerology Gustave Roussy, France

## Abstract

It has previously been shown that nano-meter sized vesicles (30–100 nm), exosomes, secreted by antigen presenting cells can induce T cell responses thus showing the potential of exosomes to be used as immunological tools. Additionally, activated CD3^+^ T cells can secrete exosomes that have the ability to modulate different immunological responses. Here, we investigated what effects exosomes originating from activated CD3^+^ T cells have on resting CD3^+^ T cells by studying T cell proliferation, cytokine production and by performing T cell and exosome phenotype characterization. Human exosomes were generated *in vitro* following CD3^+^ T cell stimulation with anti-CD28, anti-CD3 and IL-2. Our results show that exosomes purified from stimulated CD3^+^ T cells together with IL-2 were able to generate proliferation in autologous resting CD3^+^ T cells. The CD3^+^ T cells stimulated with exosomes together with IL-2 had a higher proportion of CD8^+^ T cells and had a different cytokine profile compared to controls. These results indicate that activated CD3^+^ T cells communicate with resting autologous T cells via exosomes.

## Introduction

Exosomes are secreted membrane vesicles of nanometer size (30–100 nm) formed by inward budding of late endosomes resulting in the formation of multivesicular bodies (MVBs) in the cell and subsequent release into the cytosol by exocytosis. These mechanisms were first described in the 1980s by the groups of Stahl and Johnstone when studying the maturation of erythrocytes [1], [2]. Since then exosomes have been shown to be released by several cell types including epithelial cells [3], dendritic cells [4], [5], B cells [6], T cells [7], mast cells [8], [9] and tumor cells [10], [11] among others. The presence of exosomes has also been shown in human body fluids such as plasma [12]–[13], urine [14], breast milk [15], [16], bronchoalveolar lavage [17] and malignant effusions [18]. Exosomes have been implicated in cell to cell signaling including antigen presentation [19] and RNA transfer [20]. They have also been suggested to play a role in tumor immunity both as tumor growth promoters [21] and as inhibitors of tumor growth [22].

Exosome secretion from different T cell types has been demonstrated by several groups *e.g.* from activated CD3^+^ cells [23], CD4^+^ T cells [24] and CD8^+^ T cells [25]. The exosomes from CD4^+^ T cells have been suggested to deliver antigen specific signals [24], atherogenic signals [26] and co-stimulatory signals [23] whereas exosomes from CD8^+^ T cells have been associated with non-cytotoxic suppression of HIV1 transcription [25]. While many studies have demonstrated the impact of immune signaling from exosomes derived from antigen presenting cells [5], [27], [28], [29] on T cells, not many, to our knowledge, have demonstrated the role of T cell exosome communication with other T cells. However, it has been shown that activated human T cells can release microvesicles containing Fas and APO2 ligand [30].

The cytokine IL-2 (interleukin-2) is a potent lymphokine which regulates immune responses. It stimulates the proliferation and differentiation of activated immune cell *e.g.* T cells, B cells, monocytes and natural killer cells [31]–[32]. T cells, activated by T cell receptor engagement with an antigen together with co-stimulation, are the main IL-2 secreting cells which stimulate proliferation of themselves in an autocrine manner as well as other neighboring antigen activated T cells [33]. Since activated T cells are known to secrete exosomes [24] the aim of this study was to determine if exosomes secreted from activated CD3^+^ cells could play a role in an immunological response, enhanced by exogenous IL-2, by conveying signals from their secreting cells to resting CD3^+^ cells in an *in vitro* autologous setting. We show that upon stimulation, CD3^+^ T cells from human donors secrete exosomes, and that these exosomes together with IL-2 generate an immune response in resting autologous CD3^+^ T cells. With automated cell counting, a proliferation assay, flow cytometry and a human cytokine array, we could monitor the immune response in the stimulated CD3^+^ T cells.

## Materials and Methods

### Ethics Statement

This study, conducted at Sahlgrenska Academy in Sweden, includes blood from buffy coats obtained from the blood bank at Component laboratory at Sahlgrenska University Hospital, Gothenburg, Sweden. Ethics approval was not needed since the buffy coats were provided anonymously and could not be traced back to a specific individual. This is in line with Swedish legislation section code 4§ 3p SFS 2003:460 (Lag om etikprövning av forskning som avser människor).

**Figure 1:**
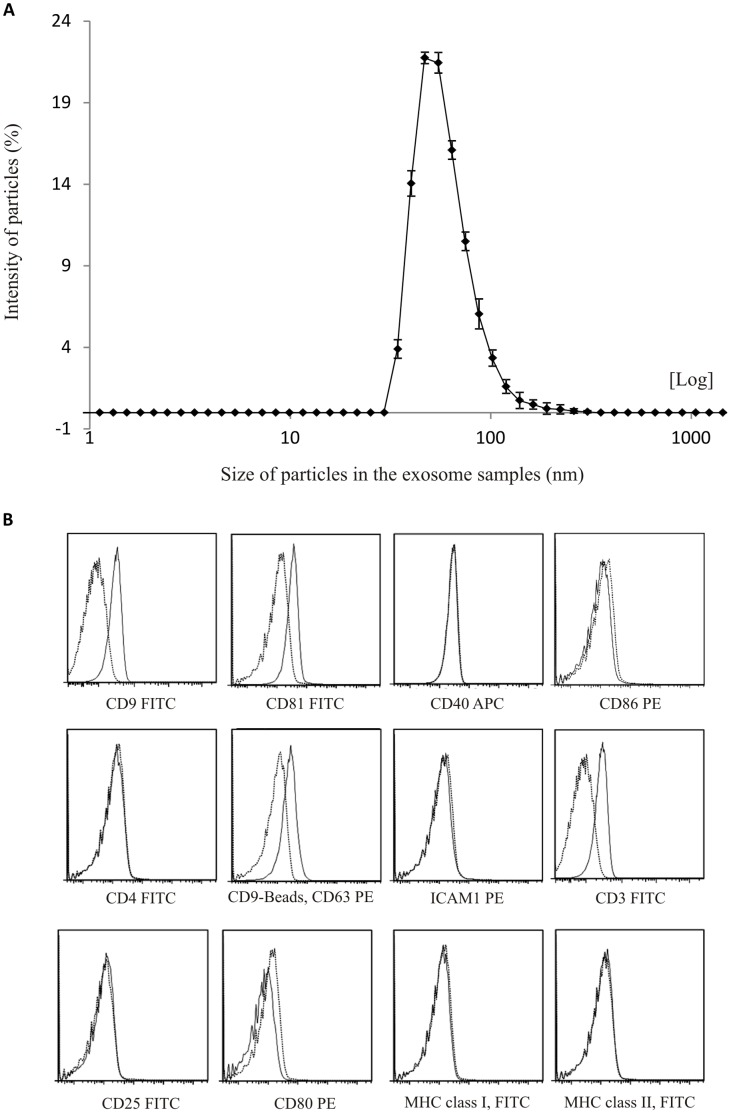
Characterization of exosomes from CD3^+^ T cells stimulated with IL-2, anti-CD3 and anti-CD28. (A) Particle sizes in ultracentrifuge pellet consistent with size range of exosomes. Average exosome size was 54 nm. Measured with dynamic light scattering (B) Exosomes bound to latex beads and stained with antibodies against exosome associated proteins (CD9, CD63 andCD81) and T cell associated proteins (CD3, CD4, CD25, CD40, CD80, CD86, MHC-I, MHC-II and ICAM-1) measured with flow cytometry. Dotted line represents isotype control.

### Cells

CD3 positive T cells were derived from peripheral blood mononuclear cells (PBMCs) from buffy coats from healthy donors (Component laboratory Sahlgrenska University Hospital, Gothenburg, Sweden) by Lymphoprep™ gradient centrifugation (Axis-Shield Poc As, Norway). Isolation of the T cells was performed using Dynabeads® Untouched™ Human T cells Kit according to manufacturer’s instructions (Dynal, Invitrogen, Sweden). The isolated cells were maintained in RPMI1640 supplemented with 10% foetal bovine serum (FBS), depleted from exosomes by ultracentrifugation at 120000×g for 70 min, 100 µg/mL streptomycin/penicillin, 2 mM L-glutamine and 1 mM sodium pyruvate (Sigma-Aldrich, Sweden) at 37°C, 5% CO_2_.

**Figure 2:**
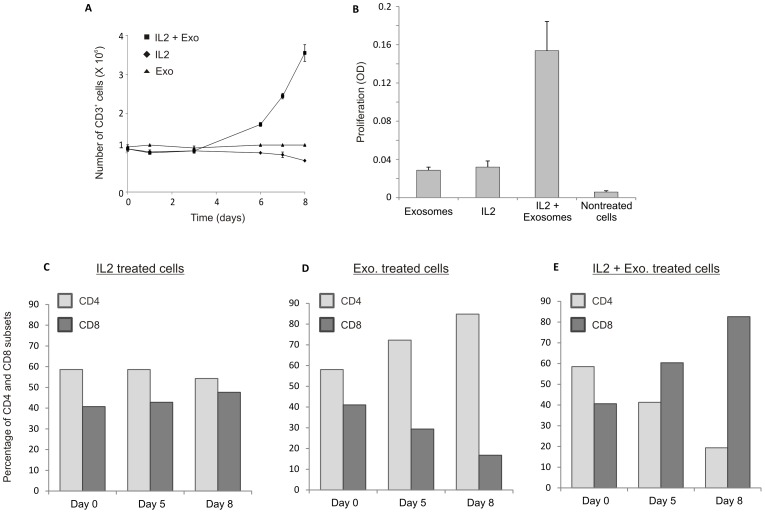
Proliferation of CD3^+^ T cells pulsed with autologous exosomes. (A) Number of CD3^+^ T cells measured with automatic cell counting (Sysmex) at different time points. Cells stimulated with IL-2+exosomes are increasing in numbers. (B) Proliferation of CD3^+^ T cells measured with MTT assay at day five. Cells stimulated with IL-2+exosomes showed increased proliferation. (C) Distribution of CD4^+^ and CD8^+^ cells in CD3^+^ T cells stimulated with IL-2, autologous exosomes or IL-2+exosomes measures with flow cytometry.

### Isolation of T cell Exosomes

To generate exosomes from CD3^+^ T cells 1×10^6^ cells/ml were incubated with 3 µg/ml anti-human CD28 (clone CD28.2), 1 µg/ml anti-human CD3 clone HIT3a (pre-coated for 2 hours at 37°C before seeding of cells) purchased from BD Biosciences Pharmingen (Belgium) and 20 ng/mL interleukin (IL)-2 (R&D Systems, UK). The supernatant was harvested after four days and exosomes were isolated by centrifugation and filtration steps as previously described [20]. Briefly, supernatants were centrifuged at 400 g for 10 min to pellet cells and at 16500×g for 30 minutes with subsequent passing through a 0.2 µm filter to remove cell debris, finally exosomes were pelleted by ultracentrifugation at 120000×g for 70 minutes in a Beckman Optima L-100 XP ultracentrifuge using a Ti70 rotor (Beckman Coulter, Germany). Exosome pellets were resuspended in Dulbeccós PBS.

**Figure 3:**
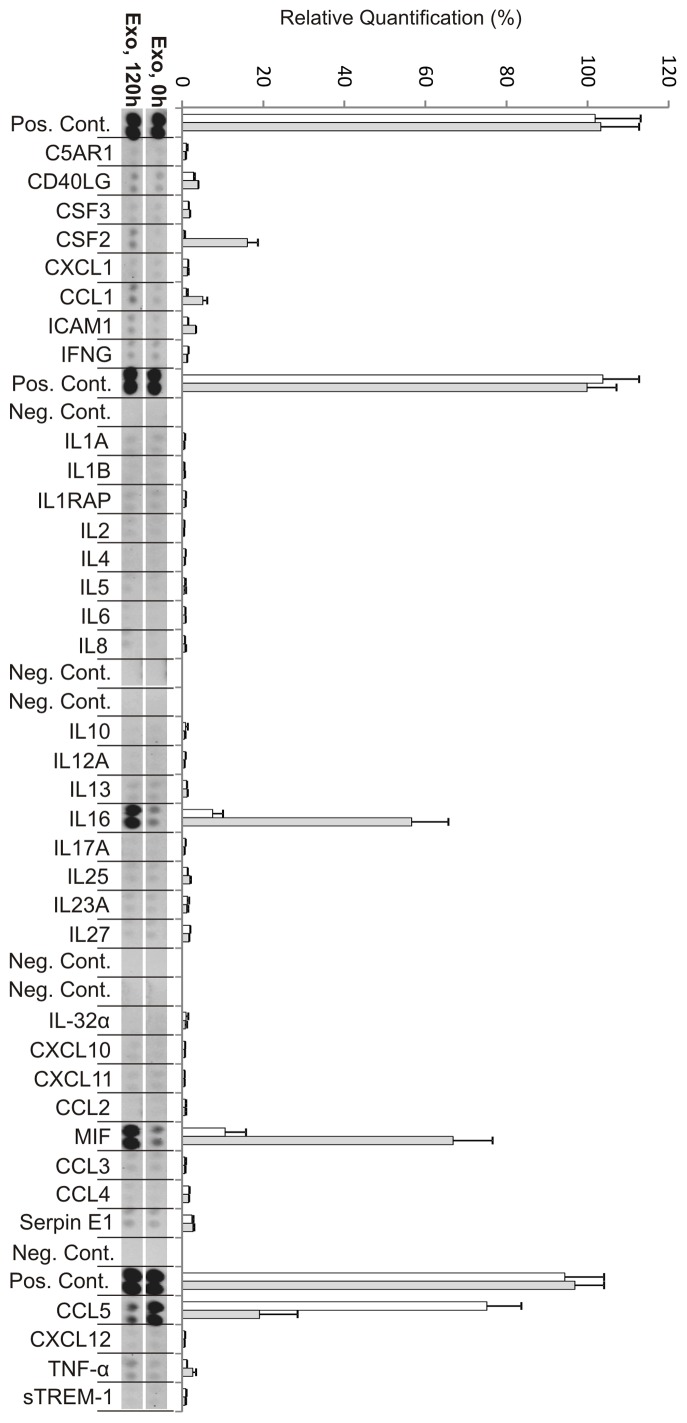
Cytokine production from autologous exosome stimulated CD3^+^ T cells at day zero (0 h) and day five (120 h). Relative quantification of spot intensities was performed using Quantity One software (Bio-Rad). Each bar represents an average of the intensity from two protein spots. White bars represent 0 h and grey bars represent 120 h (day 5). The exosomes appeared to contain significant amounts of CCL5 (RANTES) immediately after the addition of exosomes (at 0 h) since the supernatants showed relatively large amounts of RANTES. These levels were decreased at day five.

**Figure 4:**
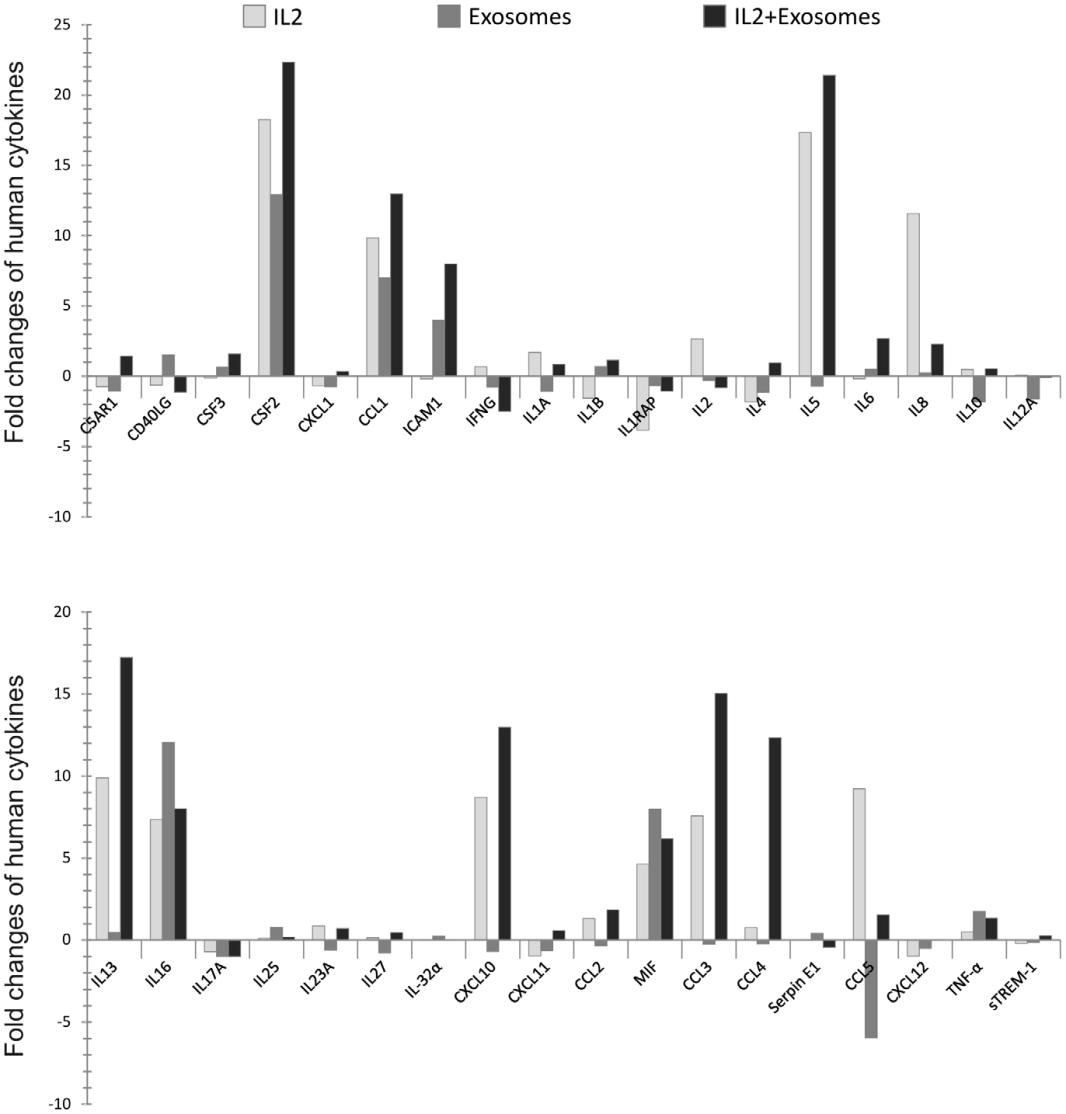
A comparison of cytokines and chemokines present in the supernatant of CD3^+^ T cells pulsed with IL-2, exosomes or IL-2+exosomes. Fold changes in the production of cytokines, chemokines and other proteins after five days. T cells stimulated with IL-2 or exosomes had different expression of cytokines and chemokines. Samples stimulated with “exosomes+IL-2″ generated secretion of more cytokines and chemokines compared to samples stimulated with either IL-2 or exosomes alone. A significant decrease could be noticed for CCL5 in cultures stimulated with exosomes only.

**Table 1:** Human cytokine array (Cytokines).

Cytokine	Alternate Name						
		IL-2		Exosomes		IL-2+Exosomes	
		Up-reg.	Down-reg.	Up-reg.	Down-reg.	Up-reg.	Down-reg.
**IL-1α**	IL-1F1	2x			1x	1x	
**IL-1β**	IL-1F2		2x	1x		1x	
**IL1RA**	IL-1F3		8x		1x		1x
**IL-2**	TCGF	3x			0.3x		1x
**IL-4**	BCGF-1		2x		1x	1x	
**IL-5**	EDF	17x			1x	21x	
**IL-6**	BSF2		0.1x	0.4x		4x	
**IL-10**	CSIF	0.5x			2x	0.5x	
**IL-12A**	CLMF-	0			2x	0	
**IL-13**	–	10x		0.4x		17x	
**IL-16**	LCF	7x		12x		8x	
**IL-17**	IL17A		2x		2x		1x
**IL-17E**	IL-25	1x		2x		0.2x	
**IL-23A**	–	1x			1x	1x	
**IL-27**	IL-27-A	1x			1x	1x	
**IL-32α**	–	1x			1x	1x	
**GM-CSF**	CSF2	18x		13x		22x	
**G-CSF**	CSF3		0,1x	1x		2x	
**IFN-γ**	Type II IFN	1x			1x		2x
**TNF-α**	TNFSF2	3x		3x		3x	
**MIF**	GIF, DER6	5x		8x		6x	
**CD40 ligand**	CD154		1x	2x			1x

Fold changes (increase/decrease) in production of **cytokines** after 5 days in CD3^+^ T cells incubated with IL-2, Exosomes, or IL-2+Exosomes.

**Table 2:** Human cytokine array (Chemokines).

Chemokines	Alternate Name						
		IL-2		Exosomes		IL-2+Exosomes	
		Up-reg.	Down-reg.	Up-reg.	Down-reg.	Up-reg.	Down-reg.
**CCL1**	I-309	10x		7x		13x	
**CCL2**	MCP-1	1x			0.4x	2x	
**CCL3**	MIP-1α	8x			0.3x	15x	
**CCL4**	MIP-1β	1x			0.2x	12x	
**CCL5**	RANTES	9x			6x	2x	
**CXCL1**	GROα		1x		1x	0.3x	
**CXCL8**	IL8	12x		0.2x		3x	
**CXCL10**	IP-10	9x			1x	13x	
**CXCL11**	I-TAC		1x		1x	1x	
**CXCL12**	SDF-1	5x		0.5x		0	

Fold changes (increase/decrease) in production of **chemokines** after 5 days in CD3^+^ T cells incubated with IL-2, Exosomes, or IL-2+Exosomes.

**Table 3:** Human cytokine array (Other proteins).

Proteins	AlternateName						
		IL-2		Exosomes		IL-2+Exosomes	
		Up-reg.	Down-reg.	Up-reg.	Down-reg.	Up-reg.	Down-reg.
**sICAM-1**	CD54		0.2	4x		8x	
**C5a**	ComplementComponent 5a		1x	1x		1x	
**Serpin E1**	PAI-1	0		0.4x			0.4x
**sTREM-1 (Receptor)**	soluble Triggering Receptor Expressed on Myeloid cells-1		1x		1x		1x

Fold changes (increase/decrease) in production of other proteins after 5 days in CD3^+^ T cells incubated with IL-2, Exosomes, or IL-2+Exosomes.

### Size Determination

Size determination of isolated exosomes was performed with dynamic light scattering (DLS) using a Zetasizer Nano ZS (Malvern Instruments, UK) according to the manufacturer’s instructions.

### Stimulation of CD3^+^ T cells

Non-stimulated CD3^+^ T cells were plated in 6- or 24-well plates (Sarstedt, Sweden) at a density of 1×10^6^ cells/ml for proliferation, flow cytometry and cytokine production assays. Immediately after seeding of CD3^+^ T cells, wells were pulsed with exosomes resuspended in PBS to a final concentration of 47 µg/ml, and/or 20 ng/mL of IL-2 (R&D Systems, UK). All stimulations were set up in duplicates. Stimulated CD3^+^ T cells were incubated for eight days. Counting of cells was done at several time points using a Sysmex K21-N cell counter (Sysmex Corporation, Kobe, Japan).

### Cell Proliferation Measurement

Thiazolyl blue tetrazolium bromide, MTT assay (Sigma-Aldrich, Sweden) was used to measure cell proliferation according to manufacturer’s instructions. Briefly, cells were treated with MTT at a final concentration of 0.5 mg/ml for 3 h at 37°C, leaving the mitochondrial dehydrogenases of viable cells to cleave the tetrazolium ring. Solubilisation solution was added overnight to dissolve the MTT formazan crystals. Absorbance was measured at 570 nm at day five. Any increase in cell number results in an increase in MTT formazan and thus in absorbance.

### Exosome Bead Coupling and Flow Cytometry

Aldehyde/sulfate latex beads (4 µm, Invitrogen, Sweden) were incubated with purified anti-CD63 clone H5C6 or purified anti-CD9 clone M-L13 (BD Biosciences Pharmingen, Belgium) overnight at room temperature with gentle agitation according to manufacturer’s instructions. The beads were blocked with 100 mM glycine (Sigma-Aldrich, Sweden) and washed with PBS with 3% FBS before incubation with exosomes. Exosomes were incubated with non-coated, anti-CD63 or anti-CD9 coated latex beads in 80 µl PBS for 15 min at room temperature. Volumes were made up to 400 µl and incubated 3 hrs at room temperature on a rotator. To block remaining binding sites, exosome coated beads were incubated for 30 min with 100 mM glycine (Sigma-Aldrich, Sweden). After two washes in PBS with 3% FBS, exosome coated beads were stained with FITC- or PE-conjugated CD81, CD9, CD63, CD3, CD4, MHC-1, MHC-II, CD25, CD86, CD80, ICAM-1 antibody or corresponding isotype control (BD Biosciences Pharmingen, Belgium).

For flow cytometry analysis of stimulated CD3^+^ cells, the cells were harvested and washed followed by staining for cell surface markers using antibodies against CD3-PerCP clone SK7, CD4-FITC clone RPA-T4 and CD8-PacificBlue clone RPA-T8 (BD Biosciences Pharmingen). All flow cytometry data were collected on a FACSCantoII (Becton, Dickinson, USA) and analyzed with FlowJo software version 7.6.3 (Tree Star, Inc, USA).

### Cytokine Measurement

Supernatants were harvested at day 0, immediately after addition of exosomes, and at day 5 from CD3^+^ T cells stimulated with IL-2 alone, exosomes alone or IL-2+exosomes. The culture supernatants were centrifuged for 5 min at 15700×g to remove cell debris and particles. Protein concentrations of the supernatants were determined by DC Protein Assay (Bio-Rad, Sweden). Analysis of cytokines in the supernatant was carried out using Proteome Profiler™Array, Human Cytokine Array Panel A (cat#ARY005, R&D Systems Europe) according to manufacturer’s instructions. The supernatants were sonicated for 5 minutes in a 65°C water bath to release exosome proteins. Volumes corresponding to 1.5 mg protein were diluted and mixed with a cocktail of biotinylated detection antibodies. The mix was incubated with the array membrane to allow cytokine antibody complexes in the sample to bind to anti-cytokine antibodies captured on the membrane. After washing away unbound material a streptavidin-HRP complex was added for detection of the antibody-protein complexes on the membrane. Detection of array spots was performed using Amersham ECL-Prime reagents (GE Healthcare Life Sciences, VWR Sweden). Chemiluminescence was measured with Molecular Imager ChemiDoc XRS system. Quantification of the intensity of the spots was made using Quantity One software (Bio-Rad).

## Results

### Characterization of Exosomes from Stimulated CD3^+^ T cells

We first investigated the potential presence of exosomes in supernatants from CD3^+^cells stimulated with CD3 and CD28 antibodies together with IL-2. Exosome isolation was performed as previously described [34] [16] [20]. The size of pelleted structures was determined with dynamic light scattering (DLS) using a Zetasizer Nano. The results showed that the pellet consisted of particles with an average size of 54 nm in diameter consistent with characteristic size range of exosomes (Figure 1A). The isolated exosomes stained positive for the canonical exosome markers CD81, CD63 and CD9 using flow cytometry (Figure 1B). Moreover, the presence of T cell specific proteins as well as other immune associated proteins was examined on the exosomes using flow cytometry. The results showed that the vesicles were additionally positive for CD3 (Figure 1B), previously reported by Tumne et al [25], but not for CD4, CD40, ICAM-1, MHC-I, MHC-II, CD80 or CD25 (Figure 1B).

### Exosomes together with IL-2 Generate Proliferation in Autologous CD3^+^ T cells

To assess whether exosomes could stimulate autologous resting T cells, the cells were pulsed with exosomes and incubated for eight days. Proliferation was analyzed by automated cell counting at determined time points (Figure 2A). Since the automated cell counting did not discriminate between live and dead cells the proliferation was also measured by MTT assay at day six (Figure 2B). The addition of exosomes only or IL-2 only, resulted in a marginal T cell proliferation (Figure 2A–B), but stimulation of the T cells with exosomes together with IL-2 induced a distinctive cell proliferation (Figure 2A–B).

### T cell Cultures Pulsed with Exosomes and IL-2 Showed a Larger Proportion of CD8 Cells after Five Days

The distribution of CD4^+^ and CD8^+^ cells within the stimulated CD3 positive cells was investigated by flow cytometry at three time points (Figure 2 C–E). Prior to stimulation, all samples had a comparable distribution with an approximate 60/40 ratio between CD4^+^ and CD8^+^ cells. IL-2 stimulated cells preserved an almost even distribution of CD4^+^ and CD8^+^ positive cells (Figure 2C). However, T cells treated with autologous exosomes show a relative increase of CD4^+^ cells and a decrease in CD8^+^ cells at all time points (Figure 2D). Interestingly, the CD3^+^ cells stimulated with exosomes together with IL-2 showed an opposite pattern with a relative increase of CD8^+^ cells and a decrease of CD4^+^ cells at day five and even more pronounced at day eight (Figure 2).

### Cytokine Profiles of Stimulated T cells

We further studied if the stimulation of CD3^+^ T cells with IL-2 only, exosomes only and exosomes together with IL-2 resulted in different cytokine profiles in the supernatants. Using a human cytokine array, we examined the presence of cytokines, chemokines and other proteins detectable within the array in the supernatants after five days.

### Exosomes together with IL-2 Generate a Different Cytokine Production in T cells Compared to Exosomes or IL-2 alone

#### Cultures stimulated with exosomes only

Samples stimulated with exosomes alone for five days showed significant production of macrophage migration inhibitory factor (MIF), IL-16 and granulocyte-macrophage colony-stimulating factor (GM-CSF), GM-CSF is also known by the alternate name CSF2 (Figure 3, Table 1). Interestingly the level of IL-16 was higher in these cultures compared to cultures stimulated with IL-2 only or exosome together with IL-2. Furthermore there was a low but significant production of the chemokine CCL1 (Figure 3). When comparing fold changes of the cytokines present at high level, GM-CSF, IL-16, and MIF were increased more than eight-fold at day five (Figure 4, Table 1). The chemokine CCL1 had a seven-fold increase at day five compared to day 0 (Figure 4, Table 2). However, a notable difference was seen in exosome stimulated cells where the exosomes as such seemed to contain significant amounts of CCL5 (RANTES) i.e. immediately after the addition of exosomes (at 0 h) the supernatants showed relatively large amounts of RANTES. These levels gradually declined and, showed a more than five times decrease at day five (Figure 4 and Table 2). The two proteins tumor necrosis factor (TNF) -α and intercellular adhesion molecule 1 (ICAM1) were present but at low levels and showed a more than two-fold increase (Figure 4 and Table 3).

#### Cultures stimulated with IL-2 only

**Figure 5:**
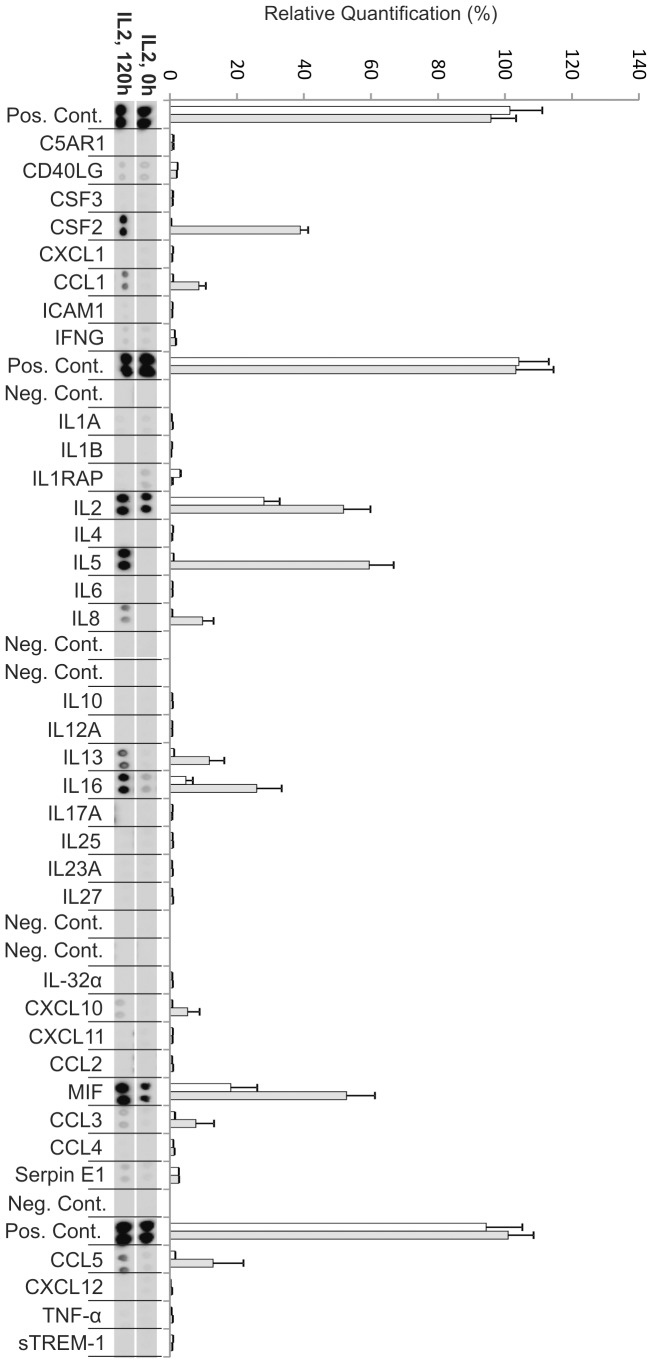
Cytokine production from IL-2 stimulated CD3^+^ T cells at day zero (0 h) and day five (120 h). Relative quantification of spot intensities was performed using Quantity One software (Bio-Rad). Each bar represents an average of the intensity from two protein spots. White bars represent 0 h and grey bars represent 120 h (day 5). Cytokines IL-5, MIF, and GM-CSF (CSF2) were present at a high level in the supernatant after five days.

After five days the cytokines IL-5, MIF, and GM-CSF were present at a high level in the supernatant from the IL-2 stimulated cells (Figure 5), where the biggest fold change could be observed for GM-CSF and IL-5 (Figure 4 and Table 1). The cytokines IL-16, IL-13, IL-8 and the chemokines CCL5, CCL1, CCL3 and CXCL10 were present at lower levels (Figure 5). These cytokines (Table 1) and chemokines (Table 2) were more than two-fold increased at day five compared to day zero (Figure 4, Table 1–2). Only one significant fold decrease could be detected in IL-1RA, which was generally present at very low levels (Figure 4, Table 1). It was not fruitful to compare the IL-2 levels since IL-2 was added at 0 h to the culture. (Figure 4, Table 1).

#### Cultures stimulated with exosomes together with IL-2

**Figure 6:**
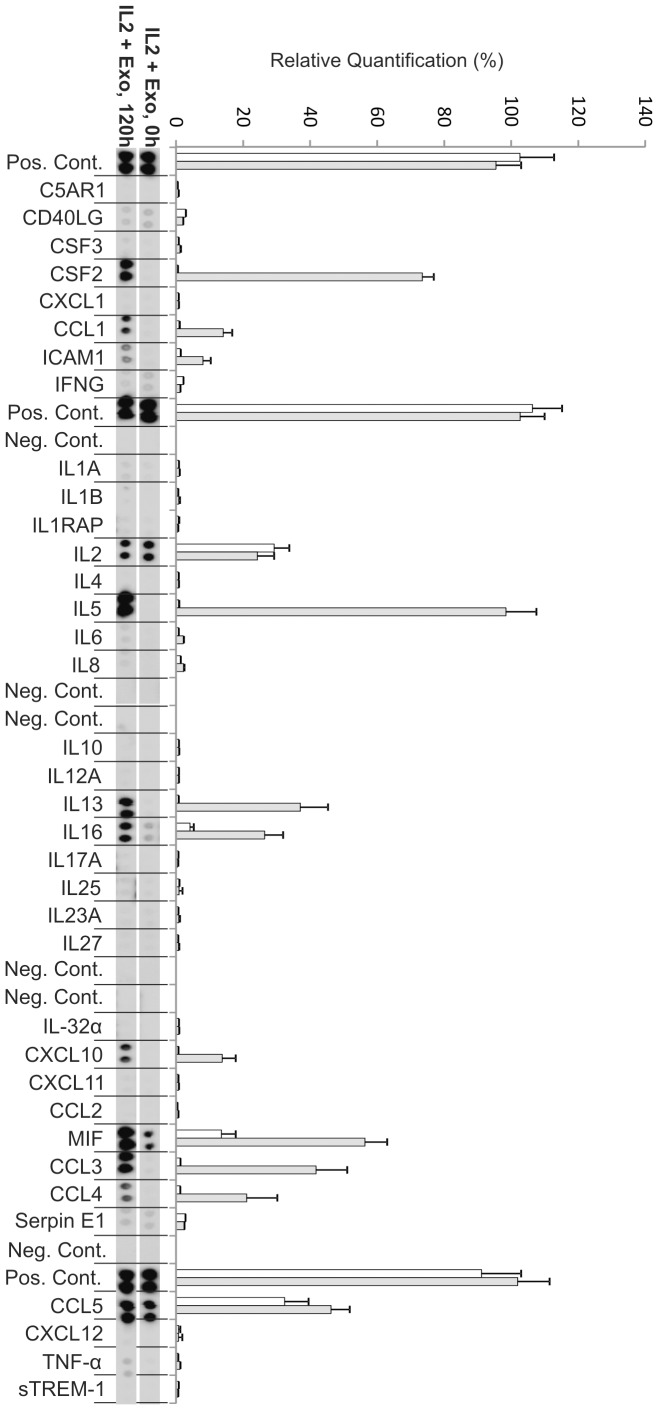
Cytokine production from exosome+IL-2 stimulated CD3^+^ T cells at day zero (0 h) and day five (120 h). Relative quantification of spot intensities was performed using Quantity One software (Bio-Rad). Each bar represents an average of the intensity from two protein spots. White bars represent 0 h and grey bars represent 120 h (day 5). The cytokines IL-5, IL-13 and GM-CSF as well as the chemokines CCL3 and CCL4 were present at higher levels at day five.

The resting T cells stimulated with exosomes together with IL-2 showed increased proliferation and a cytokine production profile at day 5 which clearly differed from cells stimulated with IL-2 or exosomes only (Figure 2B, Figure 6). In the exosome+IL-2 stimulated cells the cytokines IL-5,IL-13 and GM-CSF as well as the chemokines CCL3 and CCL4 were present at higher levels at day five (Figure 6). Interestingly CCL4 could not be detected in supernatant from cells stimulated with either exosomes or IL-2 alone. CCL1, CXCL10 and ICAM1 were all present at low levels at day 5 but not at all at day 0 which resulted in big fold increase (Figure 4, Table 2–3). As in the exosome only and IL-2 only cultures the biggest fold changes were observed for MIF (Figure 4, Table 1).

## Discussion

In this study, we show that exosomes derived from stimulated T cells can function as an autologous signal to increase proliferation of resting T cells. In addition, stimulation of resting T cells with these exosomes also results in an altered cytokine profile and in a lower CD4/CD8 ratio.

When performing studies involving exosomes it is of great importance to characterize the exosome vesicles well. In this study, using dynamic light scattering, we show that the ultracentrifuge pellets of supernatants from the primary T cells contain vesicles with an average size of 54 nm in diameter, corresponding to the size of exosomes. Since dynamic light scattering detects the size of all particles present it indirectly gives an estimate of the purity of the preparation. In addition, using flow cytometry, we show that exosome vesicles present in the supernatant from IL-2, anti-CD3 and anti-CD28 stimulated T cells have canonical exosomal markers CD9, CD63 and CD81 on their surface [35]. These results show that CD3^+^ T cells from healthy donors stimulated with anti-CD3, anti-CD28 and IL-2 secrete exosomes. This result is in line with previous studies where T cells stimulated with PHA, IL-2 and anti-CD3 produce exosomes [24].

In order to understand the role of these exosomes from activated T cells in an autologous setting, the vesicles from proliferating CD3^+^ cells were isolated and transferred to autologous resting CD3^+^ cells. To measure the effect of the exosomes on the resting CD3^+^ cells we performed proliferation assays, flow cytometry to look at CD4/CD8 ratio and a cytokine array on the cell supernatant. We show that T cell derived exosomes take part in the stimulation and proliferation of resting CD3^+^ T cells. Neither IL-2 nor exosomes can on their own stimulate the resting T cells to grow significantly. However, the autologous exosomes derived from stimulated T cells appear to cooperate with IL-2 to orchestrate proliferation of CD3^+^ T cells. Furthermore, the distribution between the CD4^+^ and CD8^+^ populations were skewed in co-cultures when exosomes from activated T cells were added. The results show that exosomes together with IL-2 result in more CD8^+^ than CD4^+^ cells at day 5 and day 8, which suggest the proliferating cells to be CD8^+^ T cells. Exosomes together with IL-2 induced a relative increase of CD8^+^ cells similar to that of IL-2 together with anti-CD3 and anti-CD28. This could infer that the exosomes carry with them information from the cells that secrete them, which can stimulate a proliferative response in resting T cells. It has previously been described that modified exosomes from APCs could induce a CD8^+^ T cell response in an antigen-specific manner [36]. Here we get a CD8^+^ T cell response probably not related to antigenic stimuli since the exosomes derive from autologous stimulated CD3^+^ T cells.

When examining the level of cytokines and chemokines present in the supernatant from the T cell cultures, we note significant changes when exosomes are present. Exosomes together with IL-2 generate secretion of more cytokines and at higher levels than exosomes or IL-2 alone. The cytokine analysis also shows that T cells stimulated with IL-2 together with exosomes secrete CCL4. This chemokine is not present in IL-2 alone or exosome alone stimulated cells but is found in the supernatant from which the exosomes are derived. This may indicate that the exosomes carry information that together with IL-2 can induce resting T cells to respond similar to the cells that secreted them. The chemokine CCL4, also known as macrophage inflammatory protein 1β, has previously been shown to be secreted by CD8^+^ T cells [37]–[38] which supports the notion of a preferential stimulation of cytotoxic T cells as also indicated by the shift in CD4/CD8 ratio. The cytokine profile of cells stimulated with exosomes+IL-2 reveals a high level of IL-5 and IL-13, which may indicate a Th2 skewing of the activated cells [39]. In addition the exosomes seem to carry with them large amounts of CCL5 (RANTES), since the addition of exosomes to the culture media makes this cytokine immediately readily detectable (Fig. 3, 0 h). Interestingly RANTES has been shown to be secreted by activated CD8^+^ T cells from a specific storage compartment with exosome properties [40] and exosomes carrying RANTES can actively inhibit HIV infection [25]. There is also a previous report demonstrating that RANTES is present in CD8^+^ cytotoxic cell granules and that it can act as a mitogen upon cell surface aggregation followed by secretion of MIP-1β [41]. These results correspond well with our observations.

In summary, our result show that exosomes secreted from simulated CD3^+^ T cells can dramatically change the response of resting autologous T cells to IL-2. The exosomes carry RANTES and seem to favor a cytotoxic response, which could be of potential interest in anti-viral and anti-tumor treatment.
